# What routine mental healthcare data could be used to predict response in major depression treatment?

**DOI:** 10.1192/j.eurpsy.2026.10581

**Published:** 2026-07-31

**Authors:** H. F. Wiegand, F. Uhl, S. Hütter, J. Stoffers-Winterling, O. Tüscher, K. Lieb, L. P. Hölzel

**Affiliations:** 1 Psychiatry and Psychotherapy, University Medicine Halle, Halle; 2 Psychiatry and Psychotherapy; 3 University Medicine Mainz, Mainz; 4 Psychiatry, Psychotherapy and Psychosomatics, University Medicine Halle, Halle, Germany

## Abstract

**Introduction:**

Routine mental health care in Europe generates a large amount of data that to date has not much been used for guiding treatment decisions: On the one hand, data primarily generated for billing purposes (care-related data, routine health insurance data), such as ICD-10 diagnosis codes, information on hospital stays and interventions or outpatient medication prescriptions. On the other hand, specific clinical data. Examples include data from Access to Psychological Therapies (IAPT) services or the Mental Health Services Data Set (MHSDS) in Great Britain, the National Health Data System (SNDS) in France or the extension module Mental Health of the German Medical Informatics Initiative’s core data set, which is currently under development.

**Objectives:**

Which variables should be included in these datasets for a reliable treatment response prediction in routine unipolar Major Depression (MD) treatment?

**Methods:**

Umbrella review of individual participant data (IPD) meta-analyses on predictors and moderators in the treatment of MD, compliant with the PRISMA-IPD statement. Mapping of results to routine mental health datasets.

**Results:**

From the 1548 articles identified by the systematic literature search, 25 eligible studies were included. The review identified predictors and moderators in the areas of depression history and course, comorbidities, sociodemographic factors, psychosocial factors, and aversive life events. The method used did not identify any biological or imaging markers as predictors or moderators. Most of the identified variables could be approximated using routine data sets, but in many cases only using complex indicators.

**Conclusions:**

Available routine data sets seem to be interesting for treatment response prediction in routine care, but in many cases complex indicators would have to be constructed. The clinical benefits would have to be tested in studies. Using available routine data would offer the opportunity to apply the results of therapy response prediction research in routine practice.

**Disclosure of Interest:**

None Declared

